# Surface expression and limited proteolysis of ADAM10 are increased by a dominant negative inhibitor of dynamin

**DOI:** 10.1186/1471-2121-12-20

**Published:** 2011-05-17

**Authors:** Robyn M Carey, Jan K Blusztajn, Barbara E Slack

**Affiliations:** 1 Department of Pathology and Laboratory Medicine, Boston University School of Medicine, 715 Albany Street, L808, Boston MA 02118 USA

**Keywords:** a disintegrin and metalloprotease (ADAM)10, dynamin, amyloid precursor protein (APP), muscarinic receptor, protein kinase C (PKC), endocytosis

## Abstract

**Background:**

The amyloid precursor protein (APP) is cleaved by β- and γ-secretases to generate toxic amyloid β (Aβ) peptides. Alternatively, *α*-secretases cleave APP within the Aβ domain, precluding Aβ formation and releasing the soluble ectodomain, sAPPα. We previously showed that inhibition of the GTPase dynamin reduced APP internalization and increased release of sAPPα, apparently by prolonging the interaction between APP and α-secretases at the plasma membrane. This was accompanied by a reduction in Aβ generation. In the present study, we investigated whether surface expression of the α-secretase ADAM (a disintegrin and metalloprotease)10 is also regulated by dynamin-dependent endocytosis.

**Results:**

Transfection of human embryonic kidney (HEK) cells stably expressing M3 muscarinic receptors with a dominant negative dynamin I mutant (dyn I K44A), increased surface expression of both immature, and mature, catalytically active forms of co-expressed ADAM10. Surface levels of ADAM10 were unaffected by activation of protein kinase C (PKC) or M3 receptors, indicating that receptor-coupled shedding of the ADAM substrate APP is unlikely to be mediated by inhibition of ADAM10 endocytosis in this cell line. Dyn I K44A strongly increased the formation of a C-terminal fragment of ADAM10, consistent with earlier reports that the ADAM10 ectodomain is itself a target for sheddases. The abundance of this fragment was increased in the presence of a γ-secretase inhibitor, but was not affected by M3 receptor activation. The dynamin mutant did not affect the distribution of ADAM10 and its C-terminal fragment between raft and non-raft membrane compartments.

**Conclusions:**

Surface expression and limited proteolysis of ADAM10 are regulated by dynamin-dependent endocytosis, but are unaffected by activation of signaling pathways that upregulate shedding of ADAM substrates such as APP. Modulation of ADAM10 internalization could affect cellular behavior in two ways: by altering the putative signaling activity of the ADAM10 C-terminal fragment, and by regulating the biological function of ADAM10 substrates such as APP and N-cadherin.

## Background

The pathogenesis of Alzheimer's disease (AD) is due, at least in part, to the formation in brain of insoluble protein deposits known as amyloid plaques. The principal component of the plaques, a neurotoxic, self-aggregating peptide known as amyloid β (Aβ), is generated by successive cleavage of the amyloid precursor protein (APP) by β- and γ-secretases. Alternatively, APP may be cleaved within its Aβ domain by α-secretases, precluding the formation of Aβ, and releasing sAPPα, a large soluble N-terminal fragment, from the cell surface. An alteration in the balance between these alternative proteolytic pathways may underlie the pathogenic process in AD [1-3]. Recent research has identified the ADAM (a disintegrin and metalloprotease) family of zinc metalloproteases, particularly ADAM10 and ADAM17/TACE (tumor necrosis factor α convertase), as α-secretases [4,5]. ADAMs cleave a variety of transmembrane proteins, including APP, within their extracellular domains, resulting in shedding of the ectodomain fragment [4]. Multiple receptor ligands, as well as direct activators of protein kinase C (PKC), stimulate ectodomain shedding of APP and other proteins [6]. However, phosphorylation of the cytoplasmic domain of APP does not appear to be necessary for signal-regulated shedding of APP [7,8]. Neither does truncation of the cytoplasmic domain of ADAM17 prevent upregulation of its activity toward several of its substrates, including tumor necrosis factor α, by phorbol ester [9,10]. On the other hand, signal-activated shedding of TrkA and the prion protein was reported to be at least partially dependent on phosphorylation of threonine 735 within the cytoplasmic domain of ADAM17/TACE by mitogen-activated protein kinase [11,12]. Taken together, the evidence suggests that multiple mechanisms contribute to signal-mediated upregulation of ectodomain shedding by ADAMs.

Mutation of internalization motifs within the C-terminus of APP, or removal of the C-terminal tail, increases surface expression of APP, and release of sAPPα [13,14]. Blocking the function of the endocytic protein dynamin by transfecting cells with dyn I K44A, a dominant-negative dynamin I mutant [15], exerts similar effects [16,17] suggesting that inhibition of endocytosis promotes ectodomain shedding by prolonging the interaction between APP and α-secretases on the plasma membrane. Because PKC phosphorylates dynamin, preventing it from associating with membrane phospholipids [18], we hypothesized that inhibition of dynamin function might be the mechanism underlying kinase-dependent sAPPα release. However, the PKC activator phorbol 12-myristate 13-acetate (PMA) did not affect the rate of APP internalization in HEK cells stably transfected with APP_695 _[17].

It was recently reported that ADAM10 is cleaved within its ectodomain by ADAM9 or ADAM15, generating a C-terminal fragment (CTF) that is then proteolyzed by γ-secretase [19-21]. The released intracellular domain subsequently translocates to the nucleus [19]. Thus, like APP, ADAM10 undergoes sequential two-step proteolysis to generate a fragment with potential signaling capabilities. Our results show that inhibition of dynamin-dependent endocytosis increases surface expression of mature ADAM10, and potentiates the formation of the ADAM10 CTF. Activation of PKC, or M3 muscarinic receptors, did not affect ADAM10 surface expression or CTF formation, suggesting that receptor- or PKC-mediated stimulation of APP shedding in these cells is not dependent on alterations in ADAM10 trafficking or proteolysis. Other physiological mechanisms that could regulate ADAM10 internalization, such as interactions with other cell surface proteins, have the potential to alter generation of the CTF, thereby modulating its putative signaling actions.

## Results

### Inhibition of dynamin function increases surface expression of ADAM10

**Figure 1:**
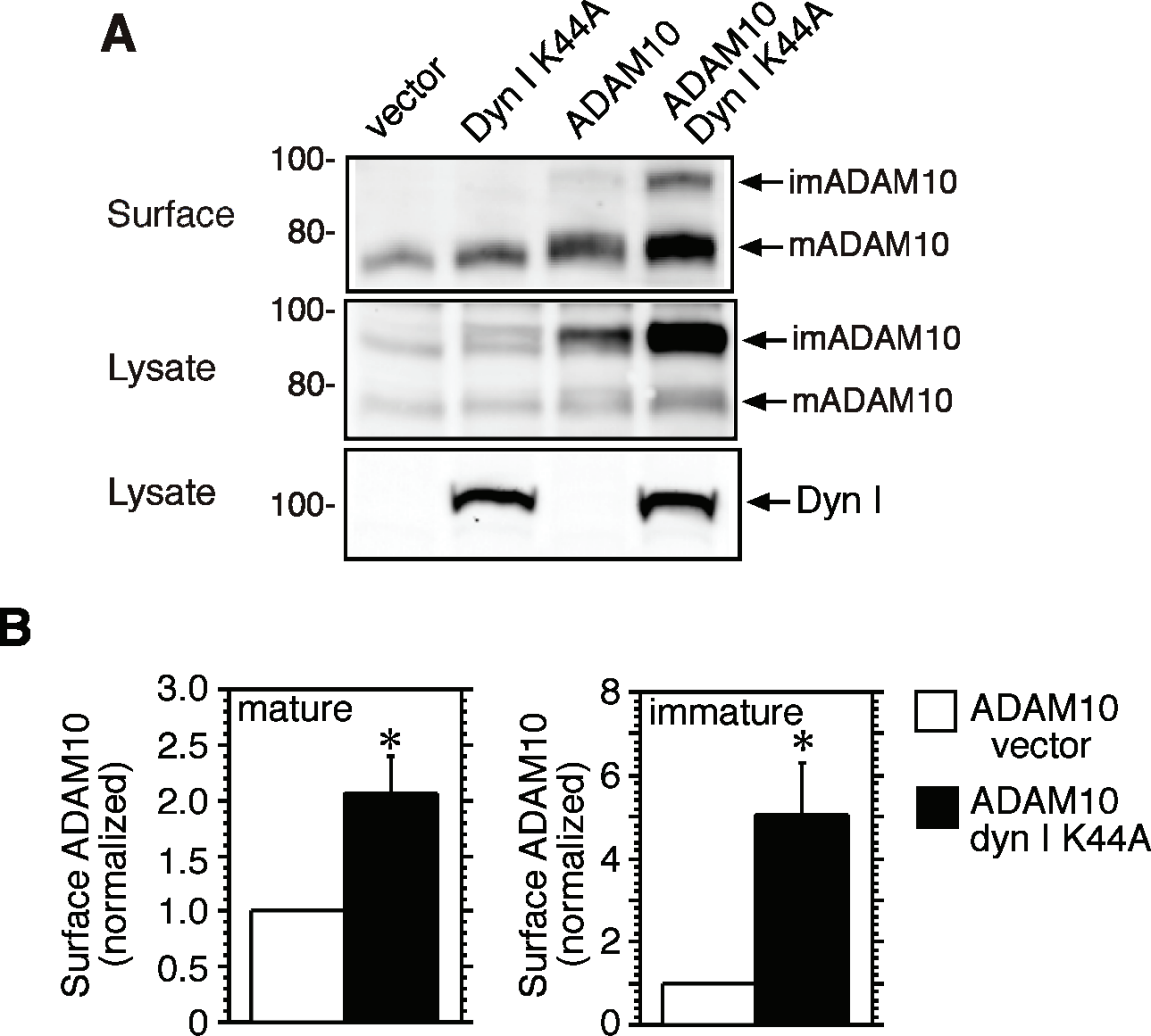
**Surface expression of ADAM10 is increased by dyn I K44A overexpression**. HEK-M3 cells were transiently transfected with ADAM10, dyn I K44A, or empty vector as indicated. After 48 h, cells were surface-biotinylated and lysed. Biotinylated proteins and cell lysates were analyzed by immunoblotting. **A**. Bands representing biotinylated immature (im) and mature (m) ADAM10 on the cell surface are indicated by arrows (upper panel). Cell lysates were immunoblotted with antibodies to ADAM10 (middle panel) and dynamin I (lower panel). **B**. Surface levels of mature and immature ADAM10 in cells co-expressing ADAM10 and dyn I K44A were quantitated and normalized relative to levels in cells co-expressing ADAM10 and empty vector. Results are expressed as means ± SEM from five experiments. *Significantly different from ADAM/vector control.

Inhibition of dynamin-dependent internalization of APP promotes its cleavage at the cell surface by α-secretases [16,17]. To determine if dynamin also regulates internalization of ADAM10, HEK-M3 cells were transiently transfected with ADAM10 and either dyn I K44A or an empty vector. After 48 h, cells were surface-biotinylated, lysed, and analyzed by Western blotting. In vector-transfected cells, biotinylated endogenous ADAM10 appeared as one band, corresponding to the mature form, which lacks the inhibitory prodomain (Figure 1A). The latter is removed by the action of proprotein convertases to generate the mature active form [22]. Overexpression of ADAM10 increased surface levels of mature ADAM10, and a second faint band representing the immature pro-form was also detected (Figure 1A, upper panel). In cells co-expressing ADAM10 and dyn I K44A, surface levels of both mature and immature ADAM10 were significantly increased compared to ADAM10/vector-transfected cells (Figure 1A, upper panel, and 1B). Mature ADAM10 at the cell surface was greatly enriched relative to the immature form of the protein; the reverse of the pattern observed in cell lysates (Figure 1A, middle panel). Interestingly, immature ADAM10 on the cell surface accumulated in the presence of the dynamin mutant to a greater degree than the mature form (Figure 1B). Although only cells overexpressing ADAM10 displayed the immature form on the cell surface, the results suggest that both mature and immature ADAM10 are internalized in a dynamin-dependent fashion.

**Figure 2:**
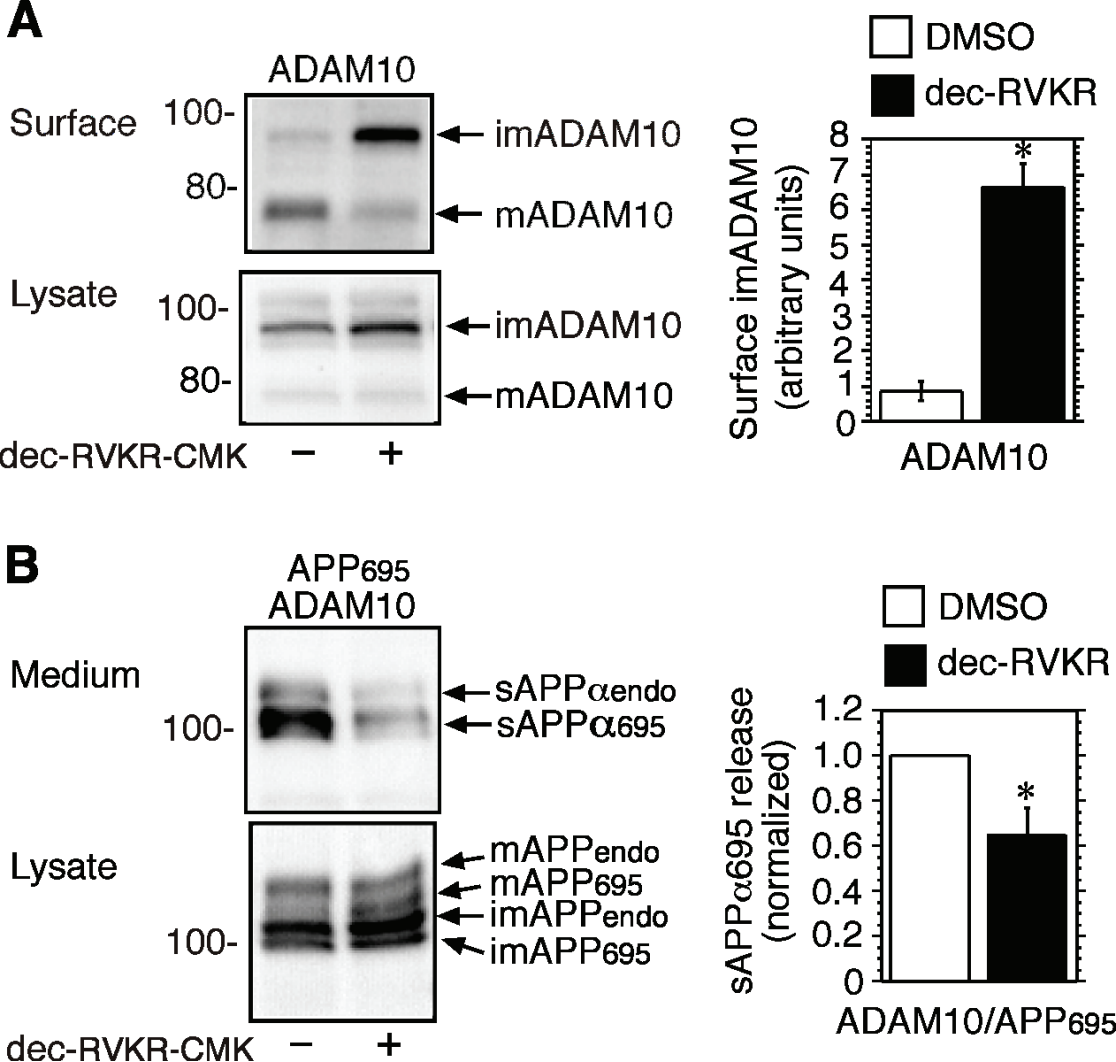
**A proprotein convertase inhibitor increases surface expression and cellular levels of immature ADAM10**. **A**. HEK-M3 cells were transiently transfected with ADAM10. After 48 h, cells were pre-treated with DMSO or the proprotein convertase inhibitor, dec-RVKR-CMK, for 2 h. Cells were surface-biotinylated and lysed. Biotinylated proteins and cell lysates were immunoblotted with antibodies to ADAM10. Levels of biotinylated immature ADAM10 were quantitated and expressed as means ± SEM from five experiments. *Significantly different from control group. **B**. Cells co-transfected with ADAM10 and APP_695 _were treated with DMSO or dec-RVKR-CMK for 2 h. Fresh serum-free medium with DMSO or dec-RVKR-CMK was then placed on the cells and the medium was collected 2 h later. Medium extracts were immunoblotted with 6E10 antibodies, and lysates with antibodies to the APP C-terminus. Levels of sAPPα_695 _in the medium were quantitated, normalized to control values, and expressed as means ± SEM from 5 experiments. *Significantly different from control values.

To confirm the identity of the anti-ADAM10-immunoreactive bands observed in surface-biotinylated isolates, cells co-transfected with ADAM10 and either APP_695 _or empty vector, were treated with a competitive inhibitor of proprotein convertases, decanoyl-arg-val-lys-arg-chloromethylketone (dec-RVKR-CMK) [22,23], or DMSO. In cells transfected with ADAM10, the inhibitor increased levels of the immature form of ADAM10 on the cell surface, and in cell lysates (Figure 2A). The release of both endogenous sAPPα, and co-transfected sAPPα_695_, was concomitantly reduced in the presence of the inhibitor (Figure 2B). This is consistent with a reduction in mature active ADAM10, and with inhibition of endogenous ADAMs, including TACE, by dec-RVKR-CMK, and is in agreement with previously published reports [22,24].

### ADAM10 overexpression increases constitutive, but not M3 receptor-evoked release of sAPPα

**Figure 3:**
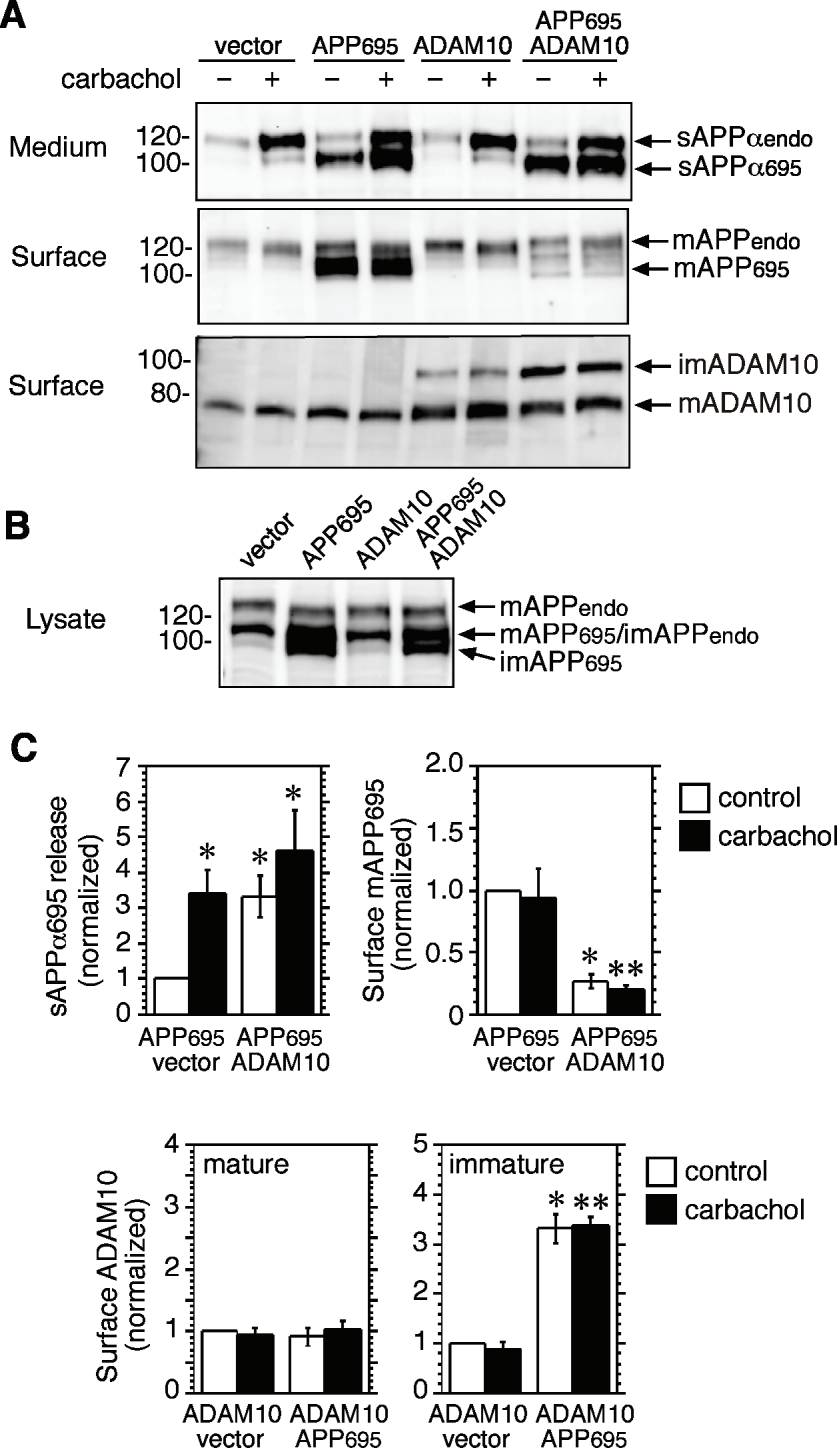
**Activation of muscarinic M3 receptors increases constitutive release of sAPPα, but does not affect ADAM10 surface expression**. **A**. HEK-M3 cells were transfected with ADAM10, APP_695_, or empty vector as indicated. After 48 h cells were pre-incubated for 1 h in serum-free DMEM, then incubated in fresh DMEM in the presence of carbachol (100 *μ*M) or vehicle control (water) for 2 h. Medium was collected, and cells were surface-biotinylated and lysed. Medium extracts were immunoblotted with 6E10 antibodies to sAPPα. Biotinylated proteins were immunoblotted with antibodies to APP or ADAM10. **B**. Lysates from vehicle-treated cultures were immunoblotted with antibodies to the APP C-terminus. **C**. Results were quantitated, normalized to control values, and expressed as means ± SEM from 4 or 5 experiments. *Significantly different from vehicle-treated APP_695_/vector transfectants. **Significantly different from carbachol-treated APP_695_/vector transfectants.

In view of evidence that ADAM10 is preferentially activated by agents that increase calcium influx [25,26], we examined the effect of the muscarinic receptor agonist carbachol on ADAM10 surface expression and α-secretase function. Previous work from this laboratory showed that a calcium ionophore stimulates sAPPα release from HEK-M3 cells, and that carbachol-mediated sAPPα release from these cells is partially dependent on calcium [27]. In HEK-M3 cells transiently transfected with APP_695_, co-expression of ADAM10 increased basal sAPPα release, while causing no further change in carbachol-evoked release, and sharply reduced surface levels of APP (Figure 3A and 3C). Conversely, co-expression with APP_695 _increased surface expression of immature ADAM10, while leaving levels of mature ADAM10 unchanged (Figure 3A and 3C). Carbachol had no effect on surface expression of ADAM10 (Figure 3A and 3C), and, despite the ability of PKC to regulate endocytosis of multiple cell surface proteins [28,29], PMA was similarly ineffective (data not shown). Overall, these results support a role for ADAM10 as a regulator of constitutive sAPPα shedding, but suggest that neither stimulation of M3 receptors, nor direct activation of PKC, affect surface expression of ADAM10 in this cell line.

### Proteolytic processing of ADAM10 is increased by dynamin inhibition, but is unaffected by carbachol

**Figure 4:**
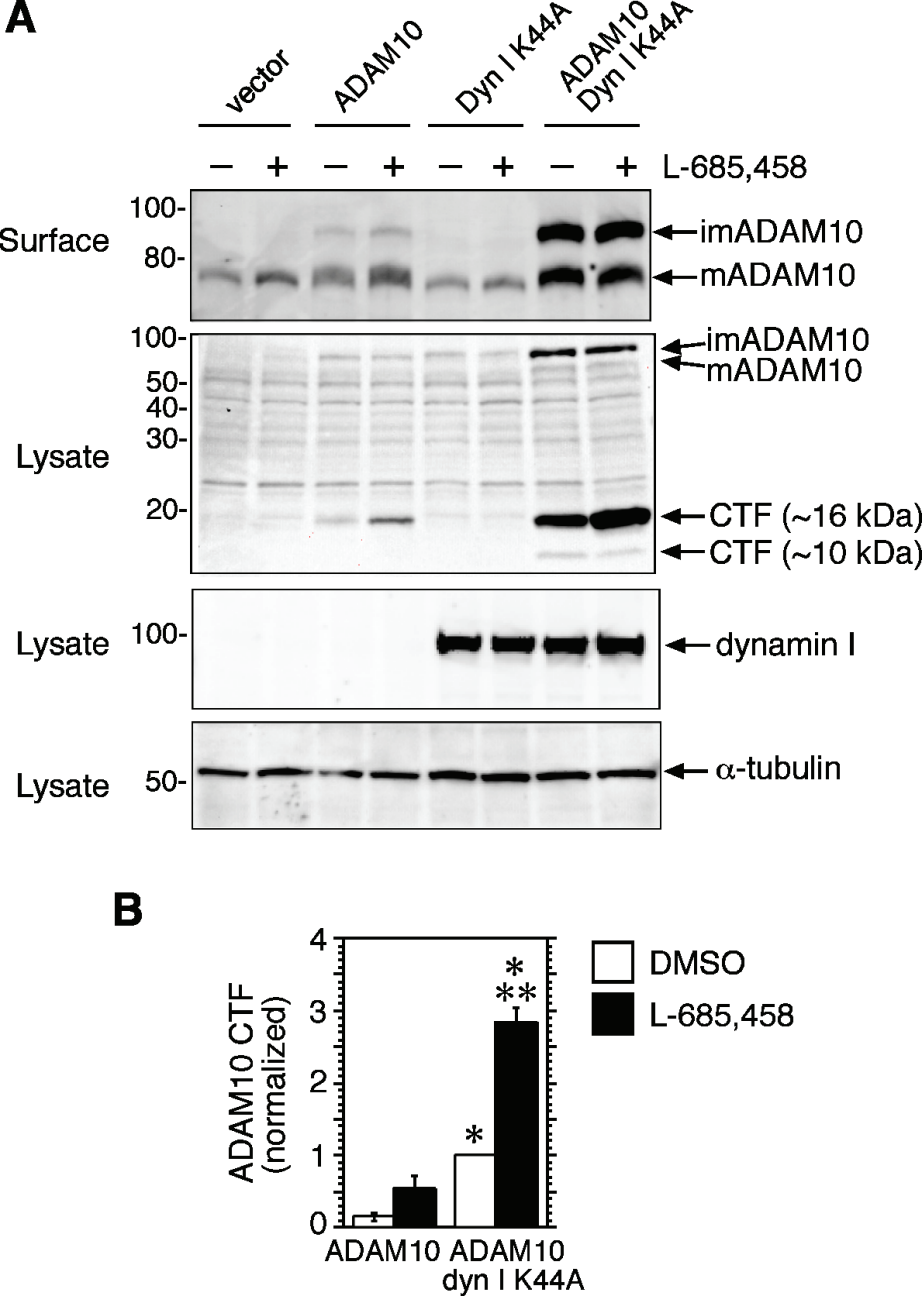
**Dyn I K44A promotes proteolytic processing of ADAM10**. **A**. HEK-M3 cells were transfected with empty vector, ADAM10, or dyn I K44A, as indicated, and allowed to grow overnight. The following day, the medium was replaced with DMEM containing 1 *μ*M L-685,458 or vehicle (DMSO). After 18 h, the cells were surface-biotinylated and then lysed. Biotinylated proteins and cell lysates were subjected to immunoblot analysis. **B**. Levels of the most prominent ADAM10 CTF (~16 kDa) were quantitated, normalized to levels in vehicle-treated ADAM10/dyn I K44A transfectants, and expressed as means ± SEM from 4 experiments. *Significantly different from corresponding ADAM10/vector transfectants. **Significantly different from vehicle-treated ADAM10/dyn I K44A-transfected cells.

**Figure 5:**
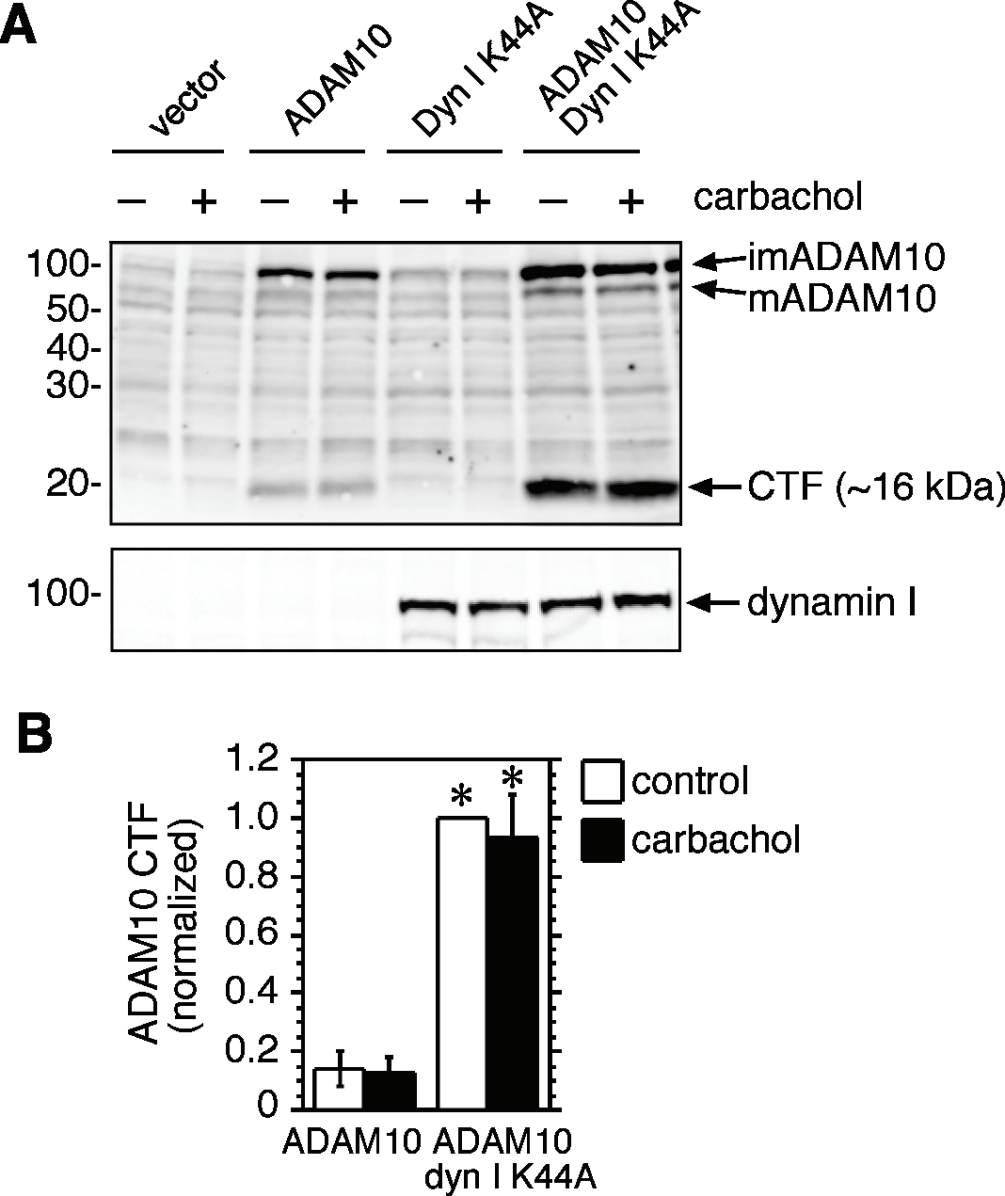
**Stimulation of M3 receptors does not accelerate formation of the ADAM10 CTF**. **A**. HEK-M3 cells were transfected with empty vector, ADAM10, or dyn I K44A, as indicated. After 48 h cells were pre-incubated for 1 h in serum-free DMEM, then incubated in fresh DMEM in the presence of carbachol (100 *μ*M) or vehicle control for 2 h. Cell lysates were prepared and subjected to immunoblot analysis. **B**. Levels of the most prominent ADAM10 CTF (~16 kDa) were quantitated, normalized to levels in vehicle-treated ADAM10/dyn I K44A transfectants, and expressed as means ± SEM from 3-4 experiments. *Significantly different from corresponding ADAM10 transfectants.

It has been reported that ADAM10 is cleaved within its ectodomain by ADAM9 or ADAM15, generating a CTF that is subsequently proteolyzed by γ-secretase [19-21]. In agreement with this study, a fragment of approximately 16 kDa was observed in cell lysates derived from HEK-M3 cells transfected with ADAM10 and probed with antibodies to the ADAM10 C-terminus (Figure 4). Levels of the CTF were increased several fold in cells co-transfected with dyn I K44A. The γ-secretase inhibitor L-685,458 significantly increased the abundance of this fragment in co-transfected cells. The inhibitor also tended to increase CTF levels in ADAM10/vector transfectants, although the effect was not statistically significant (Figure 4B). A smaller, ~10 kDa fragment was observed in ADAM10/dyn I K44A co-transfectants, but was much less abundant, and appeared to be unaffected by the inhibitor (Figure 4A). Although we were unable to detect ADAM10 immunoreactivity in the medium of ADAM10/dyn I K44A co-transfectants after a 2 h collection period, using the method described by Tousseyn et al. [19], the increased abundance of the ADAM10 CTF in lysates from cells co-transfected with dyn I K44A suggests that, like APP [17], ADAM10 proteolysis at the cell surface is increased when its internalization is inhibited. Although shedding of the ADAM10 ectodomain was reported to be increased by phorbol ester in SH-SY5Y neuroblastoma cells [20], treatment of HEK-M3 cells with carbachol had no effect on the generation of the ADAM10 CTF, either in the presence or absence of dyn I K44A (Figure 5). Under identical conditions, this concentration of carbachol, which also activates PKC in these cells [30], strongly increased APP shedding (Figure 3).

### Dynamin inhibition does not alter the distribution of ADAM10 in raft and non-raft compartments

**Figure 6:**
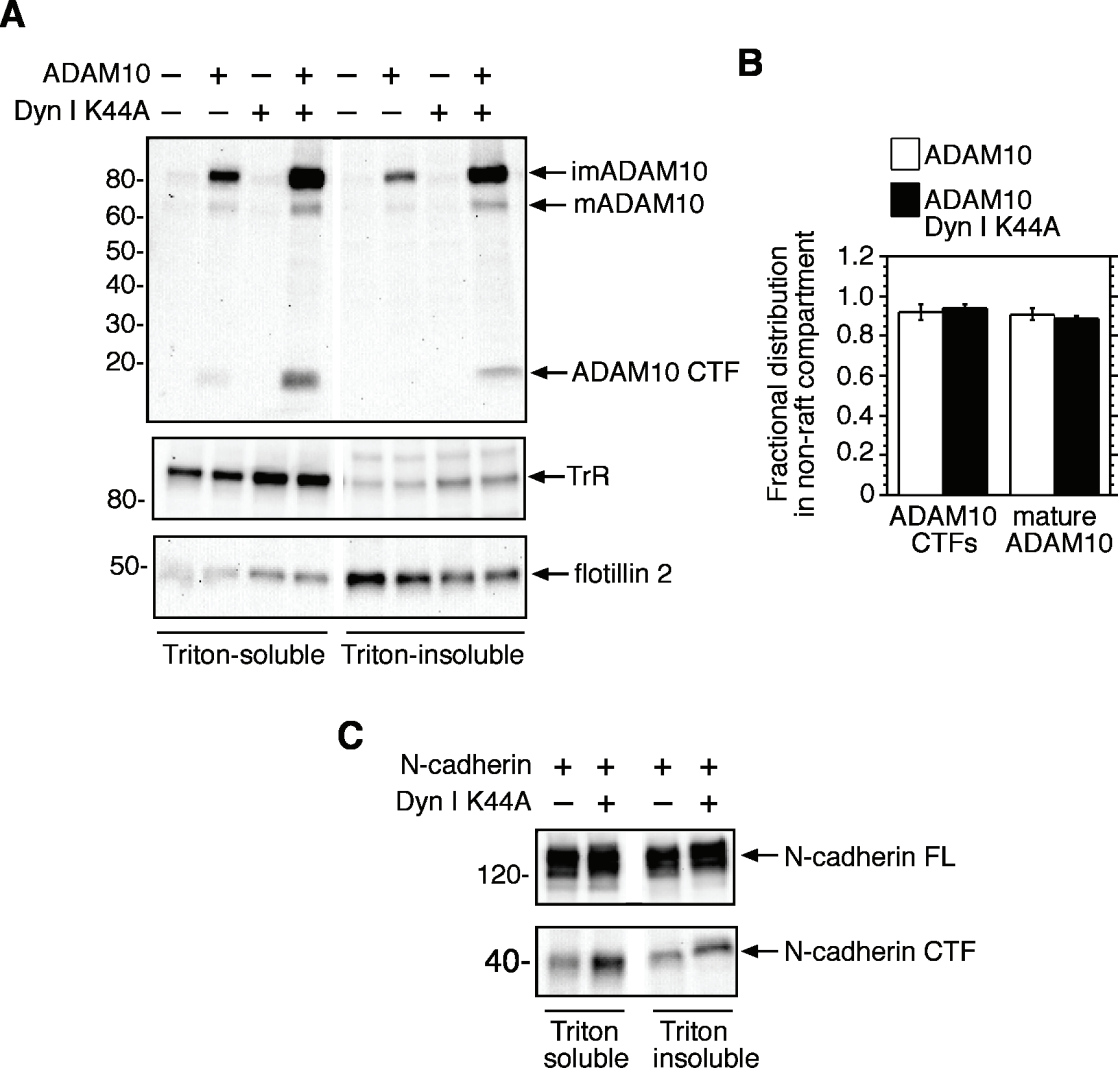
**The distribution of ADAM10 between raft and non-raft membrane regions is not affected by expression of dyn I K44A**. **A**. HEK-M3 cells were transfected with ADAM10, or dyn I K44A, as indicated. Controls received empty vector alone. After 48 h cells were fractionated into detergent-soluble and insoluble fractions. Equal amounts of protein from each fraction were size-fractionated on SDS gels and analyzed by immunoblotting with antibodies to ADAM10, the transferrin receptor (TfR) or the raft protein flotillin 2. **B**. Total ADAM10 or ADAM10 CTF levels in each fraction were calculated (band intensity × (total fraction volume/volume loaded)) and the amount in the detergent-soluble fraction was divided by the sum of the amounts in both fractions, to determine the fractional distribution of each protein in the non-raft compartment. Results are expressed as means ± SEM from 3 experiments. **C**. HEK-M3 cells were transfected with N-cadherin and empty vector, or dyn I K44A, fractionated into detergent-soluble and insoluble fractions, and analyzed by immunoblotting with antibodies to the N-cadherin C-terminus. The two halves of each blot were captured from a single image. Several unrelated lanes that appeared on the original images have been omitted for clarity.

A subset of cellular APP is located within cholesterol-rich subdomains of the plasma membrane known as lipid rafts, where it is subject to proteolysis by β- and γ-secretases [31]. In contrast, the α-secretase ADAM10 is largely excluded from lipid rafts [32]. The distribution of APP and its secretases in different membrane domains could therefore affect the balance between amyloidogenic and non-amyloidogenic processing pathways. To determine if inhibition of ADAM10 internalization affected its distribution between raft and non-raft membrane regions, we employed a modified extraction procedure utilizing Triton X-100 [33] to isolate detergent-soluble and insoluble membrane fractions from cells expressing ADAM10 and an empty vector, or dyn I K44A. Equal amounts of protein from each fraction were analyzed by immunoblotting with antibodies to the ADAM10 C-terminus (Figure 6). Full-length mature and immature ADAM10 and its CTF were detected in both detergent-soluble and insoluble fractions (Figure 6A). By expressing total protein levels in the detergent-soluble fraction as a fraction of the combined protein levels in both fractions, it was determined that approximately 90% of mature full-length ADAM10 and its CTF localized to the detergent-soluble non-raft compartment (Figure 6B). Although co-expression of dynamin I K44A increased levels of ADAM10 and its CTF in both fractions, their distribution between raft and non-raft domains was unaffected (Figure 6B). The separation of raft and non-raft domains in this experiment was indicated by the differential distribution of the transferrin receptor (TfR) and flotillin-2 in the two fractions. Approximately 97% of total TfR was localized in the detergent-soluble fraction, consistent with its designation as a non-raft marker. Flotillin is used as a marker for lipid rafts, but its relative distribution is very sensitive to the detergent used for raft isolation [34]. In our hands, 36% of total flotillin-2 was found in the detergent-insoluble fraction in ADAM10 transfectants, and 23% in ADAM10/dynamin K44A co-transfectants (n = 2). The degree of enrichment that we observed lies within the range reported by others using Triton X-100 extraction and sucrose gradient ultracentrifugation procedures [32,34].

### Dynamin regulates N-cadherin proteolysis

These results, and earlier work from our laboratory and others, indicate that dynamin regulates internalization and proteolytic processing of both ADAM10 and its substrate APP [16,17]. To extend this investigation to other transmembrane proteins, we examined the effect of the dynamin mutant on proteolytic processing of N-cadherin, an adhesion protein that is a substrate for ADAM10 and γ-secretase [35,36]. HEK-M3 cells were transiently transfected with human N-cadherin and either empty vector, or dyn I K44A, separated into detergent-soluble and insoluble fractions, and subjected to immunoblot analysis. The abundance of a ~40 kDa CTF of N-cadherin, a fragment previously shown to be a product of ADAM-mediated proteolysis [35,36], was increased in cells co-expressing dyn I K44A. The increase was more prominent in the non-raft compartment (Figure 6C). These results suggest that, as shown for APP and ADAM10, inhibition of dynamin function increases ADAM-mediated cleavage of N-cadherin, thereby promoting formation of a CTF. In the case of N-cadherin, increasing its proteolysis may potentiate its ability to act as a repressor of the transcriptional coactivator CBP (CREB binding protein) [35].

## Discussion

Cleavage of APP by the α-secretases, ADAM10 and ADAM17/TACE, is of physiological importance because it precludes the formation of the toxic Aβ peptide. Moreover, the secreted APP ectodomain fragment, sAPPα, promotes neural plasticity and neurogenesis, and exhibits neuroprotective properties [6]. G-protein coupled receptors, and compounds such as phorbol esters that directly activate PKC, increase cleavage of sAPPα by α-secretases, but the mechanism is not fully understood [6]. In the present study, we examined the effect of inhibiting dynamin-dependent endocytosis on the activity of ADAM10 toward its substrate APP.

Although other ADAMs, notably ADAM17, are capable of cleaving APP, the expression of APP in adult mouse brain overlaps to a much greater extent with ADAM10 than with ADAM17 [37]. Moreover, APP shedding is nearly abolished in primary neurons from mice null for ADAM10 [38], and in cell lines following knockdown of ADAM10 [39], indicating that ADAM10 is the physiologically relevant α-secretase in brain. Co-expression of ADAM10 with a dominant-negative dynamin mutant, dyn I K44A, increased surface expression of both mature and immature forms, indicating that endocytosis of ADAM10, like that of APP [16,17] is dynamin-dependent. An earlier report showed that endocytosis of membrane-type matrix metalloprotease 1 (MT1-MMP) is inhibited by a dynamin K44A mutant, resulting in increased cleavage of its substrate pro-MMP2 [40]. Thus, modulation of internalization could be a general mechanism regulating the activity of transmembrane metalloproteases toward their substrates.

Several laboratories have examined the effects of kinase activation on ADAM trafficking and protease activity. PMA caused down-regulation of ADAM17/TACE surface expression in Jurkat and THP-1 cells [41], and a transient increase followed by a decline in HeLa cells. The latter response was blocked by an inhibitor of mitogen-activated protein kinase kinase, and by mutation of threonine 735 to alanine of ADAM17/TACE [42]. PMA also increased degradation of the mature processed form of ADAM17/TACE, but not ADAM10, in HEK293 cells [43], and promoted the translocation of ADAM10 to the cell membrane in glioblastoma cells [44]. Taken together, the evidence indicates that PKC-regulated activation of ADAM10, or ADAM17/TACE, may be associated with alterations in trafficking in some cell types; however, neither PMA nor M3 receptor activation appear to regulate cell-surface expression of ADAM10 in HEK cells (Figure 3 and data not shown). Thus, the ability of these activators to promote APP ectodomain shedding in our model is not mediated by inhibition of internalization of either ADAM10, or its substrate APP [17].

Overexpression of ADAM10 increased constitutive, but not carbachol-evoked release of APP_695 _from HEK-M3 cells (Figure 3). Even in the absence of carbachol, surface APP_695 _was greatly reduced in ADAM10/APP_695 _co-transfectants (Figure 3), suggesting that under these conditions the response to carbachol is limited by the availability of APP_695_. This interpretation is supported by an earlier report, which showed that ADAM10 overexpression caused a much greater fold-increase in basal than in PMA-stimulated APP shedding from HEK cells, whereas a dominant negative ADAM10 mutant inhibited PMA-stimulated APP shedding by approximately 75% [45]. Interestingly, surface levels of immature ADAM10, but not mature ADAM10, were increased in cells co-transfected with APP_695_, relative to levels in cells expressing ADAM10 and empty vector (Figure 3). This suggests that the association of APP with ADAM10 inhibits the processing of ADAM10 by proprotein convertases, but how or where in the cell this occurs is unknown.

In addition to increasing surface expression of ADAM10, the dynamin mutant caused a marked elevation in cellular levels of an ADAM10 CTF, the abundance of which was further increased by the γ-secretase inhibitor L-685,458 (Figure 4). This is consistent with earlier reports that ADAM10 is subject to proteolysis by ADAM9 or 15, generating a CTF that is a substrate for γ -secretase [19-21], and suggests that the cleavage of ADAM10 occurs at the cell surface. As with surface expression of ADAM10, generation of the ADAM10 CTF was not affected by carbachol (Figure 5), in contrast with an earlier report that PMA increases ADAM10 shedding in SH-SY5Y neuroblastoma cells [20]. The reasons for this discrepancy are unclear, but could be related to cell-specific differences, which also appear to underlie the varying effects of PKC activators on ADAM10 trafficking. Because of the potential signaling role of the ADAM10 CTF [19] it would be of interest to identify factors that regulate generation of this fragment. One possible mechanism could involve alterations in ADAM10 endocytosis resulting from direct interactions with other cell surface proteins. An example of such a mechanism is the modulation of APP internalization and processing by members of the low-density lipoprotein receptor-related protein (LRP) family [46]. Notably, binding to LRP1B retains APP at the cell surface, increasing its cleavage by α-secretase, and reducing Aβ generation [46,47]. Similarly, F-spondin, a secreted signaling molecule implicated in neuronal development and repair [48], binds to both the APP ectodomain and the apolipoprotein receptor ApoE2, promoting sAPPα release, and inhibiting cleavage by β-secretase [49,50]; both these effects are consistent with reduced internalization. We predict that binding partners of ADAM10 could affect its processing in a similar way.

Evidence from multiple laboratories indicates that amyloidogenic APP processing occurs in lipid rafts, whereas processing by ADAMs is confined to non-raft regions [31]. Disruption of raft domains by depleting membrane cholesterol shifts APP processing in favor of non-amyloidogenic processing. This could reflect the displacement of APP from raft domains, and away from β- and γ-secretases [31,51], but might also be due in part to inhibition of endocytosis [52]. Although dynamin I K44A expression increased surface levels and ectodomain cleavage of mature ADAM10, there was no accompanying change in the partitioning of either full-length ADAM10 or its CTF in raft and non-raft domains (Figure 6). Thus, the effects of the dynamin mutant on ADAM proteolysis are likely simply to reflect the accumulation of ADAM10, and possibly of the ADAM that targets it, at the cell surface. To our knowledge, the effect of dynamin inhibition on the distribution of APP in membrane subdomains has not been addressed. However, like ADAM10 itself, the ADAM10 substrate N-cadherin underwent increased proteolysis in the presence of dyn I K44A, without altering its distribution between Triton-soluble and insoluble membrane domains (Figure 6C).

ADAM10 has numerous substrates [53], and alterations in its trafficking to or from the cell surface could affect its activity towards any or all of them, thereby regulating their biological activity. In the case of APP and N-cadherin, this could inhibit their ability to promote cell-cell adhesion, neuronal development and synaptogenesis [54,55], but at the same time would increase the signaling functions of their liberated C-terminal fragments [35,56]. Increased generation of the ADAM10 CTF, also a consequence of decreased internalization, might exert biological effects of its own [19], but these remain to be elucidated.

## Conclusions

Our results demonstrate that the α-secretase ADAM10 is internalized by a dynamin-dependent mechanism. Inhibition of endocytosis by co-expression of a dominant negative dynamin mutant increased surface expression of ADAM10, and promoted its proteolysis, generating a prominent CTF. Neither surface expression nor proteolysis of ADAM10 was affected by activation of muscarinic M3 receptors, despite the ability of this PKC-coupled signaling pathway to robustly stimulate APP shedding. Inhibition of dynamin also promoted cleavage of the ADAM substrate N-cadherin, but did not affect the membrane localization of either ADAM10 or N-cadherin, which were associated predominantly with non-raft domains. It will be of interest to identify physiological factors that regulate ADAM10 internalization, as these have the potential to modulate cell-cell contacts mediated by ADAM substrates such as APP and N-cadherin, and to influence the generation and signaling functions of the CTFs of both ADAM10 and its targets.

## Methods

### Materials

Antibodies and other reagents were obtained from the following sources: 6E10 antibodies to sAPPα from Signet Laboratories (Dedham, MA), rabbit antibodies to the C-terminus of APP (APP-CT) from Zymed Labs (San Francisco, CA), mouse monoclonal antibodies to dynamin I and goat polyclonal antibodies to the ADAM10 prodomain from Santa Cruz Biotechnology (Santa Cruz, CA), rabbit antibodies to the C-terminus of ADAM10 from Chemicon International (Temecula CA), and mouse antibodies to the N-cadherin C-terminus from BD Biosciences (San Jose, CA). Goat anti-mouse IgG and goat anti-rabbit IgG peroxidase-conjugated secondary antibodies were from BioRad (Hercules CA). Mouse anti-goat/sheep IgG peroxidase-conjugated antibody was obtained from Sigma. Decanoyl-arg-val-lys-arg-chloromethylketone (Dec-RVKR-CMK) and [(2R, 4R 5S)-2-Benzyl-5-(Boc-amino)-4-hydroxy-6-phenyl-hexanoyl]-Leu-Phe-NH2 (L-685,458) were purchased from Bachem Bioscience (King of Prussia, PA), Mini-gels and reagents for electrophoresis were obtained from BioRad (Hercules CA), and PVDF (polyvinylidene difluoride) membranes were purchased from Millipore (Billerica, MA). 1,10-Phenanthroline, 2-mercaptoethanesulfonic acid, carbachol (carbamylcholine chloride) and PMA were obtained from Sigma-Aldrich (St. Louis MO). Sulfo-NHS-SS-Biotin was purchased from Pierce (Rockford, IL), Other reagents and materials were acquired from Fisher Scientific (Pittsburgh PA).

### Cell culture

HEK-M3 cells (HEK cells stably transfected with M3 muscarinic receptors) were grown in Dulbecco's Modified Eagle Medium (DMEM)/F-12 supplemented with 10% fetal bovine serum (Invitrogen, Carlsbad CA) and maintained at 37°C in an atmosphere of 95% air, 5% CO_2_. The HEK-M3 cell line has been extensively characterized in our laboratory with respect to both M3 receptor signaling and APP metabolism [17,30,57,58].

### Transient transfections

Plasmids encoding the following proteins were used for transient transfections: APP_695 _(a gift from Dr. Carmela Abraham), dyn I K44A (a gift from Dr. Marc Caron), HA-ADAM10 (a gift from Dr. Falk Fahrenholz) and N-cadherin (purchased from Origene Technologies, Inc., Rockville MD). Cells were transfected with the desired plasmid, or with an empty pcDNA3 vector, using LipofectAMINE Plus™ reagent (Invitrogen) according to the manufacturer's specifications. Experiments were carried out 48 h later.

### Cell surface biotinylation

HEK cells were pre-incubated in serum-free DMEM for 1 h, and then washed in PBS, pH 7.9, supplemented with 1 mM Ca^++ ^and 1 mM Mg^++^. To biotinylate surface proteins, the cells were incubated for 30 min with Sulfo-NHS-SS-Biotin (0.5 mg/ml in PBS, Pierce, Rockford, IL). Culture dishes were kept on ice and gently rocked during the incubation period. The biotin reagent was quenched by treating the cells with two 15 min washes of 50 mM glycine in PBS. Cells were rinsed again with PBS and lysed in a buffer containing 50 mM Tris-HCl (pH 7.5), 150 mM NaCl, 2 mM AEBSF (4-(2-aminoethyl) benzenesulfonyl fluoride), 1 *μ*g/ml leupeptin, 1% (v/v) nonidet P-40, 0.05% (w/v) SDS, 0.5% (w/v) deoxycholate, and 10 mM 1,10 phenanthroline monohydrate. Lysates were incubated overnight with streptavidin-coated agarose beads (Pierce, Rockford, IL) at 4°C in a rotary mixer to isolate biotin-labeled proteins. These were size-fractionated on SDS gels, and analyzed by immunoblotting.

### Immunoblot analysis

The protein content of cell lysates was measured using the bicinchoninic acid reagent (Sigma, St Louis CA). Medium was collected, cleared by centrifugation, desalted, lyophilized, and resuspended in SDS-PAGE loading buffer, as previously described [57]. Lysates were centrifuged to remove insoluble material, and diluted in 6× loading buffer. Samples were normalized for protein content and size-fractionated on 7.5%, 10-20%, or 4-20% Tris-HCl mini-gels. Proteins were transferred to PVDF membranes, blocked for 2 h in 5%-powdered milk in Tris-buffered saline with 0.15% Tween-20 for 2 h, and probed overnight with primary antibodies. Membranes were washed and incubated with goat anti-mouse IgG, mouse anti-goat IgG or goat anti-rabbit IgG peroxidase-conjugated secondary antibodies and bands were detected using an enhanced chemiluminescence reagent (Pierce, Rockford IL). Approximate molecular weights were estimated using prestained standards from both BioRad (Hercules, CA) and Invitrogen (Carlsbad, CA). Membranes were imaged on a Kodak 440CF Image Station and quantitated using Kodak 1D Image Analysis software.

### Preparation of detergent-resistant membrane fractions

Membrane fractions were prepared according to the method of Adam et al. [33]. Briefly, cells were rinsed with PBS, suspended in buffer M containing 50 mM HEPES pH 7.4, 10 mM NaCl, 5 mM MgCl2, 0.1 mM EDTA plus protease/phosphatase inhibitor cocktail (Thermo Scientific), homogenized with 12 strokes in a Potter-Elvehjem tissue grinder, and centrifuged in a refrigerated microcentrifuge at 4°C for 5 min at 500 × g to pellet nuclei. The supernatant was decanted and centrifuged at 16,000 × g for 10 min at 4°C, the pellet was resuspended in buffer A (25 mM 2-(N-morpholino)-ethanesulfonic acid pH 6.5 and 150 mM NaCl) and then combined with an equal volume of buffer A containing 2% Triton X-100 and protease/phosphatase inhibitor cocktail. The samples were incubated on a rocking platform for 60 min on ice, then centrifuged at 16,000 × g for 20 min at 4°C. Supernatants were reserved as the Triton-soluble fraction. The pellet was rinsed with buffer A, resuspended in buffer B (20 mM Tris-HCl pH 7.6, 150 mM NaCl, 60 mM β-octylglucoside and protease/phosphatase inhibitor cocktail), and incubated on a rocking platform for 30 min on ice. The samples were centrifuged at 16,000 × g for 20 min at 4°C, and the supernatants collected as the Triton-insoluble fraction. Aliquots were assayed for protein content, and the remainder was diluted with 6× gel loading buffer and used for immunoblot analysis.

### Statistical analysis

Data are reported as means ± the standard error of the mean (S.E.M). Data were analyzed by Student's t-test or by ANOVA (analysis of variance) and Fisher's Least Significant Difference test. Differences were taken to be significant at p < 0.05. Statistical analyses were carried out using Systat software version 5.2.1 (SPSS, Inc., Chicago, IL).

## Authors' contributions

RMC and BES designed and carried out experiments and performed data analysis. JKB assisted with experimental design and assay development. All authors contributed to the writing and editing of the manuscript, and have read and approved the final version.

## List of Abbreviations

Aβ: amyloid β peptide; AD: Alzheimer's disease; ADAM: a disintegrin and metalloprotease; APP: amyloid precursor protein; CTF: C-terminal fragment; dec-RVKR-CMK: decanoyl-arg-val-lys-arg-chloromethylketone; dyn I K44A: dynamin I K44A mutant; HEK: human embryonic kidney; PKC: protein kinase C; PMA: phorbol 12-myristate 13-acetate; sAPPα: secreted APP ectodomain.
